# Autophagy in Melanoma: Molecular Mechanisms and Therapeutic Perspectives

**DOI:** 10.3390/ijms27156670

**Published:** 2026-07-26

**Authors:** Dominika Stencel, Dorota Wrześniok

**Affiliations:** Department of Pharmaceutical Chemistry, Faculty of Pharmaceutical Sciences in Sosnowiec, Medical University of Silesia in Katowice, 4 Jagiellońska Str., 41-200 Sosnowiec, Poland

**Keywords:** autophagy, melanoma, cancer, oncogenesis

## Abstract

Melanoma is a malignant tumor that originates in pigment-producing cells called melanocytes. This type of cancer remains a major public health challenge due to its high metastatic potential and resistance to treatment. Autophagy is a catabolic process that enables the controlled degradation of damaged cellular organelles and unnecessary or abnormal macromolecules. Its primary function is to maintain intracellular homeostasis and cell survival. There are three main types of autophagy: macroautophagy, microautophagy and chaperone-mediated autophagy (CMA). The role of autophagy in oncogenesis is multifaceted and context-dependent—depending on the type of cancer and its stage of development. Autophagy can either promote tumor progression or act as a tumor-suppressive mechanism. Factors influencing the role of autophagy in cancer include inflammation, crosstalk with apoptosis and resistance to anticancer therapies. Current research is focused on the use of both autophagy inhibitors and autophagy inducers as potential strategies to improve the effectiveness of melanoma treatment.

## 1. Introduction

Melanoma is a malignant tumor that arises from melanocytes, the pigment-producing cells of the skin. It is one of the most aggressive and deadly forms of skin cancer and its incidence continues to increase worldwide. The prognosis for patients with melanoma largely depends on the stage of the disease at diagnosis. While early-stage melanoma is often curable by surgical excision, the prognosis for patients with advanced or metastatic melanoma remains poor despite significant therapeutic advances [1,2,3].

Melanoma development and progression are driven by the dysregulation of several key signaling pathways, including the mitogen-activated protein kinase (MAPK), phosphatidylinositol 3-kinase/protein kinase B (PI3K/AKT), and microphthalmia-associated transcription factor (MITF) pathways [2]. Current treatment strategies are surgery, immunotherapy and targeted therapy. Although these approaches have substantially improved clinical outcomes, therapeutic resistance remains a major challenge. Consequently, there is a growing interest in identifying novel therapeutic targets and strategies that can overcome treatment resistance. One promising target is autophagy, which plays a complex role in melanoma biology and therapeutic response [1,2].

Christian de Duve discovered a cellular organelle that is responsible for degradation of intracellular materials and he called it the lysosome. In 1963, this scientist first used the term “autophagy”. Autophagy means self-eating, and it is a catabolic process based on self-degeneration [4,5,6,7]. As a result of this process, intracellular proteins and organelles are degraded and recycled. There are three forms of autophagy and they depend on membrane dynamics and cargo delivery pathways: macroautophagy, microautophagy and chaperone-mediated autophagy (CMA). The goal of autophagy is to maintain intracellular homeostasis and cell survival. This process can be triggered by different factors such as differentiation, normal growth control and starvation [8,9,10]. Autophagy can also be involved in the pathogenesis of different diseases, e.g., cardiovascular, neurodegenerative, pulmonary, metabolic, infectious and cancer. The key principles of autophagy in mammalian cells are already well known. Nevertheless, the importance of this process in cancer cells is not fully understood. This process may have a suppressive effect on tumor growth and may support cancer development. The course of autophagy in cancer cells may be influenced by the stage of the tumor, environmental factors and genetic factors [11,12].

In this review, we discuss the process of autophagy and its pivotal role in the development and progression of melanoma. Due to its dual nature, autophagy can either promote or suppress tumor growth, depending on the stage of tumor development and the cellular context. We provide a comprehensive overview of the multifaceted role of autophagy in oncogenesis and summarize the current understanding of its regulation and function in melanoma. Furthermore, we discuss emerging therapeutic strategies aimed at either inhibiting or inducing autophagy as a means of overcoming melanoma-associated treatment resistance. The incorporation of autophagy-modulating agents into clinical practice has the potential to significantly enhance the efficacy of anti-melanoma therapies, representing a promising approach to improving patient outcomes and addressing a major public health challenge. Finally, we highlight current autophagy-based therapeutic strategies that are being investigated for the treatment of melanoma.

## 2. Melanoma

Melanoma is a type of cancer originating from pigment-producing cells called melanocytes. Factors contributing to its development include genetic predisposition, exposure to ultraviolet radiation, and geographical variations. It is the most aggressive form of skin cancer and is associated with the highest risk of mortality among skin cancers. Melanoma represents a significant global healthcare challenge, as currently available treatments remain insufficiently effective [2]. Data show that the incidence of the melanoma is increasing year by year, having risen dramatically over the past several decades. In 2012, melanoma accounted for approximately 230,000 cases, while in 2020, there were 324,635 new cases and 57,043 deaths due to melanoma [13,14,15,16,17].

### 2.1. Molecular Specificity, Tumor Microenvironment (TME) and Immunological Background

Approximately 40–60% of cutaneous melanomas harbor activating mutations in the BRAF gene, with the BRAF V600E mutation being the most common. These mutations result in a constitutive activation of the BRAF kinase, leading to persistent stimulation of the mitogen-activated protein kinase (MAPK) signaling pathway, which regulates cell proliferation, survival, differentiation, and apoptosis. BRAF is a serine/threonine protein kinase that functions as a MAPK kinase kinase (MAP3K) and is normally activated downstream of RAS following stimulation of cell surface receptors, such as KIT or epidermal growth factor receptor (EGFR). Activated BRAF phosphorylates and activates MEK1/2, which subsequently phosphorylate ERK1/2. Once activated, ERK1/2 translocate to the nucleus, where they regulate the transcription of genes involved in cell proliferation, survival, and differentiation. Constitutive activation of BRAF abolishes normal regulatory control of the MAPK pathway, resulting in sustained downstream signaling, reduced dependence on upstream RAS activation, enhanced cell proliferation, and increased tumorigenic potential [18,19].

The phosphatase and tensin homolog (PTEN) is a tumor suppressor protein that plays a critical role in regulating cell growth, proliferation, and survival by negatively regulating the phosphatidylinositol 3-kinase/protein kinase B (PI3K/AKT) signaling pathway. Loss or inactivation of PTEN, which occurs in approximately 10–35% of melanomas, is one of the major mechanisms contributing to resistance to BRAF inhibitors. PTEN loss results in constitutive activation of the PI3K/AKT pathway, thereby promoting cell proliferation, survival, and inhibition of apoptosis [19,20]. AMP-activated protein kinase (AMPK) is a highly conserved cellular energy sensor that monitors intracellular AMP, ADP, and ATP levels. AMPK is activated under conditions of energy stress, such as nutrient deprivation or hypoxia, when ATP levels decrease and AMP or ADP levels increase. Once activated, AMPK restores cellular energy homeostasis by stimulating ATP-generating catabolic pathways while suppressing ATP-consuming anabolic processes [21,22]. In addition, AMPK inhibits cell growth under metabolic stress and has been reported to be upregulated in melanoma, particularly in uveal melanoma. One of the principal mechanisms by which AMPK regulates cell growth is through inhibition of the mechanistic target of rapamycin (mTOR), a central regulator of protein synthesis, cell growth, proliferation, motility, and differentiation. mTOR is a key downstream effector of the PI3K/AKT signaling pathway and serves as a major negative regulator of autophagy. Under nutrient-rich conditions, active mTOR suppresses autophagy, thereby preventing the degradation of newly synthesized cellular components and promoting cellular growth and lysosomal homeostasis. Conversely, during nutrient deprivation or hypoxia, inhibition of mTOR relieves this suppression, leading to the activation of autophagy, which provides metabolic substrates required for cell survival under stress conditions. Accumulating evidence indicates that activation of the PI3K/AKT/mTOR signaling pathway is associated with the progression of melanoma from primary lesions to invasive and metastatic disease, highlighting its central role in melanoma progression and therapeutic resistance [21,22,23].

The tumor microenvironment plays a critical role throughout all stages of tumorigenesis, including tumor initiation, progression, metastasis, and therapeutic response. In addition to malignant cells, the melanoma TME comprises a variety of stromal and immune cell populations, including cancer-associated fibroblasts (CAFs), keratinocytes, adipocytes, endothelial cells, and infiltrating immune cells, as well as non-cellular components such as the extracellular matrix (ECM), blood and lymphatic vessels, cytokines, chemokines, and other signaling molecules [24,25]. The melanoma microenvironment is characterized by hypoxia, acidosis, and nutrient deprivation, all of which contribute to tumor progression and therapeutic resistance. Melanoma cells actively remodel the TME through the secretion of extracellular vesicles, particularly exosomes, which can be retained within the extracellular matrix through interactions with collagen and other ECM components. These exosomes mediate intercellular communication by transferring proteins, lipids, and nucleic acids to neighboring stromal and immune cells, thereby promoting tumor growth, invasion, angiogenesis, immune evasion, and metastatic dissemination [24,25]. The tumor microenvironment also plays a pivotal role in the development of resistance to anticancer therapy. Cellular components of the tumor niche contribute to the heterogeneity and phenotypic plasticity of melanoma, facilitating immune escape and the acquisition of therapeutic resistance. Melanoma cells can reprogram surrounding stromal cells in several ways. For example, they activate cancer-associated fibroblasts, particularly during metastatic progression, enhancing extracellular matrix remodeling, invasion, and angiogenesis. Likewise, melanoma cells can stimulate keratinocytes to secrete factors that further promote tumor invasion and progression. The phosphatidylinositol 3-kinase/protein kinase B/mechanistic target of rapamycin (PI3K/AKT/mTOR) signaling axis is also a key regulator of the tumor microenvironment. This pathway integrates signals derived from growth factors, cytokines, nutrient availability, and hypoxia to regulate immune cell recruitment, differentiation, polarization, and effector functions. Consequently, aberrant activation of the PI3K/AKT/mTOR pathway contributes to the establishment of an immunosuppressive tumor microenvironment, thereby promoting melanoma progression, metastasis, and resistance to therapy [24,26,27].

The immune system’s role is to protect against infections and diseases caused by microorganisms, with the aim of maintaining the body’s homeostasis. The immune system also works to detect and eliminate precancerous cells in order to prevent progression to melanoma. To eliminate melanoma cells, the innate immune system must quickly eliminate cancer cells and work to recruit adaptive immune cells, present tumor antigens via major histocompatibility complexes (MHCs) and provide adequate costimulatory signaling via surface receptors and/or cytokines to generate a long-term, tumor specific memory population [28]. Due to its high mutation rate, melanoma is one of the most immunogenic types of cancer. The plasticity of melanoma cells allows them to adapt to adverse conditions in the immune microenvironment. This immunogenicity results from the expression of tumor-associated antigens (TAAs), i.e., lineage-specific markers that are overexpressed in melanoma cells, such as MART-1, tyrosinase and gp100, cancer testis antigens and neoantigens. Melanoma cells can also evade detection by T cells. As data shows, effector populations, such as CD4+ T cells, CD8+ T cells, and NK cells, contribute to tumor control, whereas Tregs and myeloid-derived suppressor cells (MDSCs) suppress host immune surveillance [28,29].

Immune checkpoint proteins are also essential for maintaining immune homeostasis. They are found on the surface of cells and include CTLA-4 and PD-1, whose function is to regulate the initiation, duration, and intensity of immune responses. An example of this is the binding of PD-1 to its ligands PD-L1 and PD-L2, which leads to inhibition of T-cell activation, proliferation, and cytokine secretion, preventing self-tissue damage from excessive immune response. However, cancer cells, including melanoma cells, can overcome this defense mechanism of the organism by overexpressing PD-L1, which binds to PD-1 on cells, leading to suppression of T-cell function and ineffective recognition and killing of tumor cells. The protein that serves as a key checkpoint in the immune system is CTLA-4. It is expressed primarily on activated T cells and competes with CD28/B7 binding, thereby inhibiting T-cell activation and proliferation [30,31].

### 2.2. Diagnosis and Therapeutic Strategy

The diagnosis of melanoma remains a significant challenge. Clinical diagnosis is based on the ABCDE criteria, which refer to asymmetry, border irregularity, color variability, a diameter of 6 mm or greater, and evolution and recent change. Amelanotic melanoma is more difficult to detect because it does not meet most diagnostic criteria. Screening tests for melanoma are currently conducted, but unfortunately, their introduction has not reduced the mortality rate from this cancer [13,32,33]. There are four main stages of melanoma (I, II, III, VI), with stages I and II indicating early-stage melanoma, while stages III and IV indicate advanced-stage melanoma [13,32,33]. Currently, melanoma therapy involves tumor excision with margins determined by tumor thickness, followed by drug therapy, checkpoint immunotherapy (immune checkpoint inhibitors), targeted therapy, or radiotherapy [32]. Modern therapies have improved treatment quality, particularly for unresectable or metastatic melanoma. In recent years, the treatment and survival rates of patients with amelanotic melanoma (AM) have also improved dramatically. Treating AM is challenging because it is the solid tumor with the highest number of mutations, and cancer cells can evade the immune system. The main advantages of modern treatment methods, such as targeted therapies and immunotherapies, include greater efficacy and extended patient survival. Nevertheless, serious adverse effects are still frequently observed, which may lead to the necessity of discontinuing the treatment. These side effects include fatigue, diarrhea, rash, nausea, vomiting, fever, hyperglycemia, headache and colitis [17].

### 2.3. Therapeutic Resistance

Resistance to anticancer therapy can be intrinsic—helping tumors survive from the start of treatment and allowing them to continue progressing—or acquired—involving intracellular changes in the stromal microenvironment that allow the tumor to adapt to treatment [34]. In the case of treatment with immune checkpoint inhibitors, approximately 55% of patients with melanoma exhibit innate resistance to a single-agent PD-1 inhibitor, and nearly 25% patients acquire resistance to PD-1 inhibitor within 2 years of treatment [35]. Defects in interferon gamma (IFNγ) signaling have been identified in 4–10% of PD-1-resistant melanomas. The most common factors contributing to resistance to treatment with immune checkpoint inhibitors affect antigen presentation and likely reduce tumor immunogenicity. These changes include loss of function in the beta-2-microglobulin gene, downregulation of MHC-I expression, loss of MHC heterozygosity and silencing of wild-type melanocytic differentiation antigens (e.g., MART-1/Melan-A and gp100) [32,35]. As research indicates, certain characteristics of the tumor microenvironment and the gut microbiome are associated with the development of resistance to treatment with immune checkpoint inhibitors [35]. Mechanisms of treatment resistance include the BRAF mutation. As a result of increased RAS protein activity, BRAF signaling occurs even in the presence of BRAF inhibitors. This signaling pathway leads to the desensitization of melanoma cells to targeted therapy [2,34]. Other resistance mechanisms that lead to loss of response to treatment include secondary mutations in BRAF (e.g., gain-of-function (GOF) mutations in RAS and loss-of-function (LOF) mutations in NF1), structural changes in BRAF that are independent of mutations, activation of the MAPK pathway downstream of BRAF, as well as the upregulation and activation of other receptor tyrosine kinases (RTK). In addition, autophagy can promote tumor resistance to BRAF inhibitors [34].

Despite improvements in treatment quality and effectiveness, the disease remains incurable in about 50% of cases. The introduction of targeted therapy and immunotherapy has reduced melanoma mortality, but metastasis still remains a serious problem. Melanoma has a high propensity for rapid progression to metastasis, and it is often incurable [32,36,37]. Data show that patients with metastasis live about 6–9 months, with 3-year survival rates around 15% and a 5-year survival rate of only 4.6% [37]. Additionally, the high cost of the treatment is noteworthy. In the United States alone, annual treatment costs were about $3.3 billion. Statistics indicate that from 2011 to 2030, the cost of treating melanoma will increase by 252.4% [38]. All-above mentioned problems indicate that there is still a need to search for new therapeutic approaches to improve treatment effectiveness.

## 3. Autophagy: Basic Information

**Figure 1:**
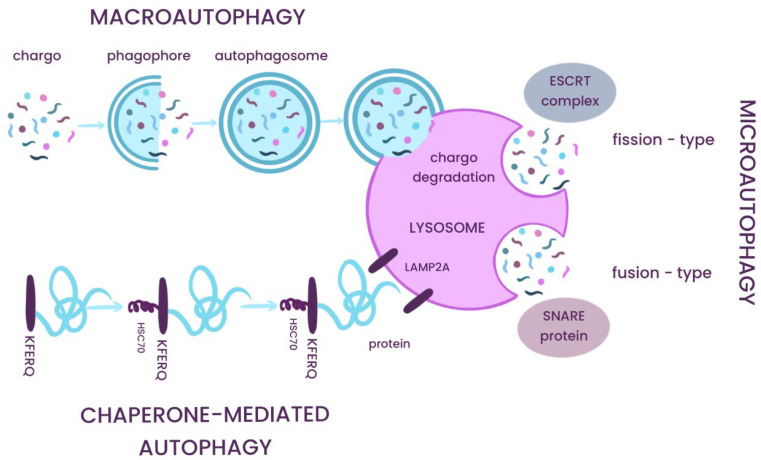
Types of autophagy. There are three main types of autophagy: macroautophagy, microautophagy, and chaperone-mediated autophagy (CMA). The specific pathway depends on the nature of the cargo being transported for degradation. In the macroautophagy, cytoplasmic cargo is enclosed within double-membrane vesicles called autophagosomes, which subsequently fuse with lysosomes, allowing the cargo to be degraded. In microautophagy, the lysosomal membrane directly invaginates to engulf cytoplasmic material, thereby delivering the cargo into the lysosomal lumen. Microautophagy can be classified into two types: a fission-type and a fusion-type. In the fission-type microautophagy, the ESCRT (endosomal sorting complex required for transport) machinery plays a crucial role by mediating membrane deformation and invagination. In the fusion-type microautophagy, membrane invaginations or protrusions are formed and subsequently sealed through fusion with phagophore-like membrane structures. This process is mediated by SNARE proteins (soluble N-ethylmaleimide-sensitive factor attachment protein receptors). In the chaperone-mediated autophagy, proteins targeted for degradation must contain a KFERQ-like motif that is recognized by the molecular chaperone HSC70 (heat-shock cognate protein 70). The chaperone–substrate complex is then transported to the lysosomal membrane, where it binds to the LAMP-2A receptor complex, facilitating the translocation of the substrate into the lysosomal lumen for degradation.

In the cells of living organisms, processes are constantly taking place to maintain intracellular homeostasis. One such process is autophagy and it can occur in cells of various organisms such as animal cells, plant cells and yeast cells. The main function of autophagy is the conserved lysosomal degradation of organelles and cytoplasmatic components. This process appears in the cells both under physiological conditions and during nutrient starvation, inflammation, microbial infections, aging and immune responses [39,40,41]. There are three main forms of autophagy: macroautophagy, microautophagy and chaperone-mediated autophagy (CMA) (Figure 1). Proteins of the ATG family play an important role in the proper progression of autophagy. These proteins are involved in different stages of this process [39,40]. The key pathways regulating the process of autophagy are mTOR and AMPK. When intracellular energy levels are sufficient, the mTOR signaling pathway inhibits autophagy. Conversely, the AMPK signaling pathway activates ULK1 (UNC-51-like kinase 1), thereby inducing autophagy [42,43].

Macroautophagy is the most extensively studied form of autophagy and is often referred to simply as autophagy. The different types of autophagy are classified according to the mechanisms by which cargo is delivered to the lysosome [39,44]. In the macroautophagy, substrates that are intended to be degraded are captured in double-membrane vesicles (phagophore) and a structure called an autophagosome is formed. Next, the autophagosome is transported to the lysosomes for degradation. The phagophore is formed with the participation of the lipid transferase ATG2 (supplies lipids from endoplasmic reticulum) and the lipid scramblase ATG9 (balances lipids across the phagophore bilayer). During this stage in the phagophore membrane, for one to a few members of the ATG8 family are conjugated to a phosphatidylethanolamine. The mammalian homologues of yeast ATG8 comprise two major subfamilies: MAP1 light chain 3 (LC3) and GABA_A_ receptor-associated protein (GABARAP). LC3 was the first mammalian homolog to be identified and has become synonymous with the autophagosome. The ATG8 homologues are crucial for autophagosome maturation. The closed and mature vesicles can fuse with the lysosome and their cargo can be degraded (Figure 1) [45,46,47].

Microautophagy does not involve the formation of an autophagosome to transport cargo to the lysosome. Instead, cargo is directly engulfed by invagination of the lysosomal or late endosomal membrane. There are different classifications of the microautophagy process, but one classification divides this process into fission-type microautophagy and a fusion-type microautophagy [48,49]. In the fission-type microautophagy, the ESCRT complex (endosomal sorting complex required for transport) is particularly important because it is responsible for membrane deformation or invagination. The mechanism of action of the ESCRT complex resembles endosomal sorting and ILV (intraluminal vesicle) formation. In the fusion-type microautophagy, membrane invaginations or extensions are generated and then sealed by fusion with phagophore-like structures. This type of microautophagy is mediated by the SNARE protein (soluble N-ethylmaleimide-sensitive factor attachment protein receptors) (Figure 1) [49,50,51].

Chaperone-mediated autophagy (CMA) does not involve vesicle formation. Instead, it is a highly selective process in which proteins destined for degradation must contain a KFERQ-like pentapeptide motif. This motif is recognized by heat shock cognate protein 70 (HSC70, also known as HSPA8), which binds the substrate protein and delivers it to the lysosomal membrane. There, the substrate forms a chaperone–substrate complex prior to translocation into the lysosomal lumen for degradation. Next, this complex combines with the LAMP2A complex located on the surface of the lysosome, allowing the translocation of the substrate to the interior of the lysosome (the HSC70 remains outside the lysosome) (Figure 1) [52,53,54,55].

Autophagy is responsible for the degradation of various cellular components. This process can be either non-selective or selective [56]. During non-selective autophagy, commonly referred to as bulk autophagy, random components of the cytosol are degraded, primarily in response to nutrient starvation. In contrast, selective autophagy targets specific substrates, such as damaged organelles, protein aggregates, and invading pathogens, including bacteria. Selectivity is achieved through the specific labeling of cargo and the action of cytosolic autophagy receptors, which recognize and deliver cargo to the autophagic machinery. Examples of these receptors include p62/sequestosome 1 (SQSTM1), NBR1, optineurin (OPTN), and nuclear dot protein 52 (NDP52). Consequently, dysregulation of selective autophagy has been implicated in the development of numerous human diseases and pathological conditions [56,57,58].

## 4. Autophagy in Cancers

Besides its crucial role in maintaining cellular homeostasis, autophagy is implicated in the pathogenesis of a wide range of diseases. As one of the most complex cellular processes, autophagy can exert dual and context-dependent effects, functioning as both a pro-apoptotic and anti-apoptotic mechanism, as well as a pro-tumorigenic or anti-tumorigenic process. Therefore, the autophagy over-activation or insufficiency can lead to the development of many diseases. Often, these diseases are associated with mutations in autophagy genes. These are neurodegenerative diseases, cardiovascular disease, autoimmune diseases, cancers and other diseases. In cancer, the protective role of autophagy promotes cancer formation and the cytotoxic role of autophagy reduces tumor progression. It depends on the stage of tumor development, microenvironment stress, pathogenic conditions and nutrient availability [12,59,60,61,62].

### 4.1. Autophagy Promoting Oncogenesis

Autophagy contributes to cancer progression by reducing the sensitivity of tumor cells to environmental and intracellular stress. Furthermore, it promotes the survival of tumor cells that enter a state of dormancy or senescence in response to anticancer therapy. In advanced cancers, autophagy supports tumor growth and progression and is associated with the development of resistance to chemotherapeutic agents. Autophagy, through the modulation of proteins and corresponding pathways, such as p53, Beclin 1 (BECN1), BIF-1 (Bax-interacting factor-1), mTOR, RAS (rat sarcoma virus), PI3KI (Class I PI3K), AKT (protein kinase B), Bcl-2 (B-cell lymphoma 2), UVRAG (ultraviolet irradiation resistance-associated gene) and BRAF supports cancer cell survival [62,63,64,65].

Under stress conditions, such as starvation and hypoxia, Beclin-1 promotes cell proliferation and spontaneous tumorigenesis, for example, in lymphomas, liver and lung carcinomas. Moreover, it has been shown that mice with one copy of the gene coding for the BECN1 interactor autophagy/Beclin 1 regulator (AMBRA1) have a higher frequency of spontaneous tumorigenesis than their wild-type littermates [37,39]. The role of autophagy in supporting cancer survival and progression influences future phases of tumor development. Additionally, the autophagy can enhance the aggressiveness of tumors by supporting metastasis and is an important process for tumors to progress to a malignant stage [62,66,67].

The immune system plays a crucial role in suppressing carcinogenesis and metastasis. Through the activity of cytotoxic T lymphocytes (CTLs) and natural killer (NK) cells, it is able to recognize and eliminate tumor cells. However, autophagy can also promote tumor progression by modulating the function of immune cells, including macrophages, which may exert pro-metastatic effects. In addition, autophagy reduces tumor cell necrosis and influences immune cell infiltration, thereby promoting the survival of tumor cells under conditions of metabolic stress and hypoxia [67,68]. Moreover, when the metastatic cells enter the blood vessels, autophagy is a factor that allows them to overcome anoikis. Anoikis is a specific type of programmed cell death (PCD) induced by detachment from the extracellular matrix, so dysregulation in this space can lead to metastasis. Autophagy, by recycling cellular components during metabolic stress, delays anoikis and sustains ATP levels until cells reattach to the matrix. Therefore, this process serves as a temporary survival mechanism. It is regulated by AMPK activation, and facilitated by AMPK/ATG6/ATG8 [69,70].

Under starvation conditions, autophagy is associated with the inhibition of cell division, motility, and protein synthesis, which leads to energy conservation. Unfavorable microenvironments and stress conditions, such as variations in the availability of nutrients, encourage cells to quiescence or activate autophagy-mediated survival pathways. Cells capable of reactivation remain dormant, but when the conditions are appropriate, they are able to resume tumor growth and proliferation, leading to disease progression. As data show, autophagy is highly active in dormant cancer cells such as ovary, breast, pancreas, gastrointestinal and bone cancers, as well as their respective mice tumor or xenograft models [71,72,73].

The tumor microenvironment is metabolically challenging because cancer cells are exposed to various stress conditions, including nutrient deprivation and hypoxia. Autophagy enables tumor cells to overcome nutrient starvation through intracellular recycling of cellular components. Consequently, autophagy has the potential to fuel nearly all aspects of central carbon metabolism by providing metabolic substrates. For example, the degradation of DNA into nucleosides can support glycolysis, while the breakdown of carbohydrates also provides substrates that fuel glycolytic metabolism. In this autophagy process, cytoplasmatic proteins, lipids, protein aggregates and organelles are delivered to lysosomes for catabolic breakdown to molecular building blocks that may be reused by the cell for macromolecular synthesis and energy. Moreover, autophagy, through its involvement in tumor metabolism, may play a role in the metabolic crosstalk between tumors and stromal cells (e.g., in pancreatic cancer) [74,75].

As demonstrated by numerous studies, there is a strong relationship between inflammation and cancer. Research has shown that autophagy also plays a pivotal role in regulating inflammation [76]. Additionally, the communication between autophagy and inflammasomes (large protein complexes required for the secretion of mature interleukin (IL)-1β and IL-18 and proptosis, an inflammatory, caspase-1-dependent form of programmed cell death) found in specific intracellular organelles may activate the tumor promotion. These interactions can influence cancer initiation and progression through various mechanisms [76,77,78]. As data show, about 20% of total cancer cases are connected with infections. For example, in the case of viruses, these are hepatitis B virus (HBV), hepatitis C virus (HCV), Epstein-Barr virus (EBV), Kaposi’s sarcoma-associated herpesvirus (KSHV), as well as potentially coronavirus 2 (SARS-CoV-2) [79,80]. These viruses can affect several molecular pathways in host cells, leading to uncontrolled proliferation, inhibition of cell death, and chronic inflammation. The mechanisms described above may contribute to the promotion of tumorigenesis. Moreover, oncogenic viruses have the ability to alter autophagy. There are known bacteria, such as *Porphyromonas gingivalis* or *Fusobacterium nucleatum*, that contribute to tumor development by inducing autophagy. In the case of the first one, the infection controls proliferation and G1 arrest in host oral cancer cells and the second infection promotes cancer by upregulating ULK1 and ATG7 to induce resistance to oxaliplatin and 5-fluorouracil in colorectal cancer [79,80,81].

One of the factors contributing to the influence of autophagy on tumor development is treatment resistance. Cellular stress induced by anticancer therapies, including chemotherapy, radiotherapy, and targeted therapy, can be reduced by autophagy, thereby decreasing the effectiveness of these treatments. These stress conditions can depend on endoplasmic reticulum stress, mitochondrial stress, oxidative stress, bioenergetic stress and genotoxic stress. Autophagy can also lead to the recycling of certain receptors, resulting in reduced efficacy of targeted therapy. Moreover, the autophagy supports cancer stem cells (CSCs) growth. There are suggestions that cancer stem cells are responsible for drug resistance [82,83,84].

### 4.2. Autophagy Suppressing Oncogenesis

The main goal of protective autophagy is to restore the cellular balance disturbed as a result of DNA damage, metabolic stress, ROS, damaged organelles, oncogenic molecules and misfolded, polyubiquitinated proteins [85,86]. There are genes, such as p53, PTEN (phosphatase and tensin homolog), TSC1/2 (tuberous sclerosis protein 1 and 2), DAPk (death-associated protein kinase) and proteins such as Beclin-1, AMBRA 1, BIF-1 and UVRAG, which are tumor suppressor factors and induce autophagy. There are known genes that activate oncogenesis, and there are autophagy-suppressing genes—RAS and AKT. As data show, in several cancers, downregulation and loss of function of some ATGs can suggest tumor inhibitory functions for autophagy [64,85]. As studies on mouse models have shown, genetic suppression of autophagic genes such as ATG 5, ATG 7 and FIP200 in connection with activation of cancer-initiating mutations leads to an increase in premalignant lesions in the GEM models of cancer. Furthermore, deletion of either ATG5 or ATG7 results in the spontaneous formation of benign hepatomas in tissues that are prone to chronic damage and inflammation [66].

Efficient autophagy can contribute to the suppression of genomic and genetic defects that may lead to oncogenesis. This process plays a protective role by reducing oxidative damage and maintaining cellular integrity during the early stages of cancer development. Therefore, autophagy prevents ROS-induced mutations, which may contribute to tumor initiation and progression. An example of this protective mechanism is the removal of dysfunctional mitochondria, which produce highly genotoxic reactive oxygen species (ROS) that can damage DNA and proteins, through a process known as mitophagy. Selective engulfment of mitochondrial cargo occurs through the action of adaptor proteins, such as BNIP3 and NIX, in combination with E3 ubiquitin ligases. Additionally, mitophagy regulators, such as the Parkin protein, encoded by the PARK2 gene, are often deleted in, e.g., melanoma. The absence of Parkin E3 ubiquitin ligase leads to the accumulation of dysfunctional mitochondria which results in an increase in ROS levels [63,65,87,88,89]. It can also decrease the cytoplasmic debris and oxidative stress, which are associated with genomic or chromosomal instabilities and the accumulation of oncogenic mutations. The role of autophagy in genomic stabilization during the early stages of tumor development includes degradation of overexpressed or misfolded proteins, mitochondrial quality control and reduction in ROS, engulfment of micronuclei and damaged nuclear parts, as well as defense against cellular carcinogenic pathogens. Therefore, defects of autophagy genes such as ATG4C, ATG5, ATG7 and UVRAG are connected with higher risk of developing malignancies. Furthermore, in cancers such as prostate, ovarian and breast cancer a reduced expression level or monoallelic deletions of BECN1 have been detected. Decreased expression of BECN1 as well as the accumulation of p62, which are the pivotal autophagy regulators, results in increased genomic instability [86,90,91,92].

Research suggests that the autophagy can inhibit necrosis and inflammation, therefore this process plays a suppressing role in the formation of neoplasia. The autophagy process contributes to numerous immune responses and helps the body fight cancers and pathogens due to its powerful ability to stimulate immunity [76]. By attenuating tumor-promoting inflammation, this process can stimulate anti-tumor immunity through enhanced processing and presentation of tumor antigens. Various signaling pathways that regulate autophagy also control tumor-associated inflammation. TLR (toll-like receptor) signaling, ROS, inflammatory cytokines and the IKK/NF-κB (IκB kinase/nuclear factor kappa-light-chain-enhancer of activated B cells) signaling axis play important roles in the interaction between autophagy and tumor-associated inflammation [50]. In natural immunity, NLR (NOD-like receptor family) and TLRs activate autophagy. This process influences macrophage differentiation and polarization. Inhibition of autophagosome degradation enhances M1 polarization which increases anti-tumor immunity [78,93]. Chronic inflammation creates a microenvironment conducive to tumor growth. The chronic inflammation can be caused by persistent infection with a pathogen (e.g., *Helicobacter pylori*—associated gastric cancer) or toxin exposure (e.g., smoking—associated lung cancer). Cancer promotion occurs in part through elevating oxidative stress and cancer-causing mutations. The autophagy process removes damaged structures, leading to a reduction in inflammation, which results in the tumor suppression. Moreover, autophagy acts as a protective mechanism that counteracts the hyperactivation of inflammasomes and the chronic inflammation associated with inflammation-induced cancer. This process can inhibit inflammasome activation by breaking down damaged mitochondria, mitochondrial reactive oxygen species and inflammasome components [78,93,94,95].

Autophagy and apoptosis are antagonistic processes whose crosstalk can lead to cooperation for cellular demise, under specific biological conditions. The crosstalk between autophagy and apoptosis contributes to tumor suppression in the early stages of cancer development [96,97]. Several key molecules are involved in the crosstalk between these processes, including Beclin-1, components of the ATG5–ATG12–ATG16 complex, p53, BIM (a BH3-only protein), Mst1 kinase, BNIP3, and the DNA mismatch repair (MMR) pathway. Moreover, there is an interaction between Beclin-1 and antiapoptotic BCL-2 proteins. Decreased BCL-2 expression may lead to excessive Beclin-1-dependent autophagy, whereas increased Beclin-1 expression can promote the release of BAK and BAX from BCL-2, thereby facilitating apoptosis. Furthermore, studies have shown that cells with elevated autophagic flux are more susceptible to FAS-induced apoptosis. This increased sensitivity is attributed to the selective autophagic degradation of FAP-1/PTPN13, a protein tyrosine phosphatase [98,99,100,101]. There are suggestions that autophagy in combination with other death signals induces the cell death process, while autophagy alone does not cause cell death. An example of this is a protein that plays a modulatory role in autophagy—DRAM1. Its signaling, together with p62 signaling, leads to cell death, which shows that autophagy is crucial but not sufficient for initiating or progression of the cell death process [64].

## 5. Autophagy in Melanoma

As is well established, autophagy exerts a dual role in cancer, including melanoma, by either suppressing or promoting tumor development, depending on the biological context. In melanoma, autophagy constitutes a primary defense mechanism against the malignant transformation of normal melanocytes. However, once tumorigenesis has been initiated, autophagy may facilitate tumor progression and contribute to therapeutic resistance under specific conditions [1].

### 5.1. Promoting Melanoma Progression in the Context of Autophagy

Autophagy plays a complex role in melanoma development and progression. Increased autophagic activity has been reported in melanoma and is associated with a poor therapeutic response. However, several studies have demonstrated an inverse correlation between the expression of autophagy-related genes and the metastatic potential of melanoma, indicating that inhibition of autophagy may enhance tumor aggressiveness and metastatic dissemination [97,102].

Monoallelic deletion or promoter methylation of ATG5, a gene encoding a protein essential for autophagosome membrane elongation, results in reduced autophagic activity in melanoma and is associated with poor patient survival, particularly in primary tumors [97,103,104]. In addition, downregulation of ATG5 expression has been shown to bypass oncogene-induced senescence in vitro, suggesting that impaired autophagy facilitates malignant transformation. Similarly, the expression levels of ATG5, Beclin 1, and LC3 are reduced in dysplastic melanocytic lesions and melanoma compared with normal melanocytes in the early stages of malignant transformation (Table 1) [103,104].

Experimental studies have demonstrated that deletion of ATG7 suppresses tumor initiation in BRAF V600E-driven melanoma, suggesting that ATG7-dependent autophagy contributes to the early stages of melanomagenesis [97]. However, in melanomas harboring both the BRAF V600E mutation and PTEN loss, the tumor-suppressive effects associated with ATG7 deficiency are abolished, indicating that the biological consequences of autophagy inhibition depend on the underlying genetic background. Furthermore, activating BRAF mutations have been associated with an increased risk of progression to advanced-stage melanoma (Table 1) [105].

Reduced expression of both ATG5 and ATG7 in primary and metastatic melanoma tissues has been attributed to deficiency of nuclear respiratory factor 1 (NRF1), providing additional evidence that dysregulation of the autophagic machinery contributes to melanoma pathogenesis [1].

Receptor tyrosine kinases (RTKs) play a pivotal role in the development and progression of BRAF-mutant cutaneous melanoma (CM). Evidence indicates that phosphorylation of the epidermal growth factor receptor (EGFR) activates Beclin-1, thereby initiating autophagy in BRAF-mutant M14 and WM793 melanoma cell lines. Another major regulator of autophagy in melanoma is the AMP-activated protein kinase (AMPK) signaling pathway. Activation of AMPK-dependent autophagy has been shown to be essential for the survival of BRAF-mutant cutaneous melanoma cells (Table 1) [106].

Accumulating evidence further suggests that BRAF V600E-targeted therapy induces autophagy in melanoma, thereby contributing to the development of therapeutic resistance. Pharmacological inhibition of BRAF has been demonstrated to stimulate cytoprotective autophagy through activation of the eukaryotic translation initiation factor 2 alpha kinase 3 (EIF2AK3/PERK)-dependent endoplasmic reticulum (ER) stress response. These findings indicate that enhanced autophagic activity promotes melanoma cell survival during BRAF inhibitor treatment, ultimately contributing to drug resistance and highlighting autophagy as a potential therapeutic target for overcoming resistance to targeted therapy [1].

Moreover, the overexpression of BECN1 gene (located on chromosome 17q21), which encodes Beclin-1, has been associated with early metastatic dissemination and a poor prognosis in uveal melanoma, likely owing to its involvement in cellular adaptation to hypoxia and acidic microenvironmental conditions. In addition, poor clinical outcome in uveal melanoma has been correlated with increased expression of BNIP1, IKBKE, ITGA6, and FKBP1A, as well as reduced expression of LMCD1, GABARAPL1, DLC1, PRKCD, and TUSC1. Elevated BNIP3 expression has also been associated with deeper scleral invasion, increased tumor pigmentation, and unfavorable prognosis in patients with uveal melanoma (Table 1) [105,107].

Signal transducer and activator of transcription (STAT) proteins are key regulators of numerous cellular processes, including immune responses, cell proliferation, differentiation, and apoptosis. Among the STAT family members, STAT6 has been shown to promote uveal melanoma progression by suppressing autophagy, thereby facilitating tumor development [108].

During the early stages of melanoma progression, the mRNA and protein expression levels of several pro-autophagic markers, including MAP1LC3A, MAP1LC3B, BECN1, and ATG5, are lower than those observed in benign melanocytic nevi. Furthermore, LC3 has been implicated in melanogenesis through regulation of microphthalmia-associated transcription factor (MITF) expression. Increased LC3B expression has been associated with highly pigmented melanoma, enhanced metastatic potential, and poor clinical prognosis, suggesting that dysregulated autophagy contributes to melanoma progression and disease aggressiveness (Table 1) [1,109,110].

Studies have demonstrated that reduced autophagic activity, in combination with the loss of functional p53, accelerates tumorigenesis. One proposed mechanism is that sublethal cellular stress resulting from ATG7 deficiency in oncogene-primed melanocytes is sensed by p53, which subsequently activates apoptotic pathways. In contrast, once melanoma has developed, tumor cells can exploit autophagy as an adaptive survival mechanism, enabling them to withstand the stressful conditions of the tumor microenvironment and thereby promoting disease progression (Table 1) [111].

Evidence also indicates that the BH3-only protein Noxa plays an important role in melanoma development and progression. Oncogenic activation of the MEK/ERK signaling pathway increases Noxa expression through the transcription factor cAMP response element-binding protein (CREB), which in turn promotes autophagy. Furthermore, Noxa has been identified as a critical mediator of MEK/ERK-driven autophagy. Under nutrient-deprived conditions, this signaling axis induces constitutive autophagic activity, thereby delaying apoptosis in human melanoma cells. Consistent with these findings, melanoma cells exposed to an acidic microenvironment exhibit enhanced autophagic activity, which facilitates their survival under adverse conditions [111].

### 5.2. Inhibiting Melanoma Progression in the Context of Autophagy

As discussed above, the role of ATG7-mediated autophagy in melanoma remains controversial. Some studies have demonstrated that inhibition of ATG7, leading to autophagy deficiency, promotes melanoma development. In contrast, other investigations employing a similar experimental model have reported opposing findings [1]. Specifically, in melanoma driven by oncogenic BRAF V600E in combination with PTEN loss, ATG7 deficiency was shown to impair both melanoma initiation and subsequent tumor progression, suggesting that ATG7-dependent basal autophagy may facilitate oncogenic transformation. These findings further indicate that autophagy is dispensable for melanoma growth but may instead function as a barrier to tumor development, a protective role that is compromised in PTEN-hemizygous animals [1,103,112]. The discrepancies observed among these studies employing similar melanoma models may be attributable to the complex interplay between autophagy, oncogenic signaling pathways, and cellular stress responses (Table 1) [1].

Other studies have demonstrated that BRAF inhibitors activate autophagy in melanoma cells by initiating a transcriptional program that promotes lysosomal biogenesis and function through activation of transcription factor EB (TFEB). In this context, increased autophagic activity is not associated with an unfavorable therapeutic response. Instead, BRAF V600E inhibitor-induced activation of TFEB has been linked to enhanced tumor regression, suggesting that the biological consequences of autophagy activation depend on the underlying molecular mechanisms and cellular context [1]. Moreover, suppression of ATG5 reduces oncogene-induced senescence in BRAF-driven melanomagenesis, further supporting the role of autophagy in limiting the initiation of malignant transformation (Table 1) [97].

Available evidence indicates that autophagic activity is elevated in approximately 40–50% of uveal melanomas, primarily as an adaptive response to tumor hypoxia. As mentioned, BECN1 overexpression may be associated with early metastatic spread and a poor prognosis in patients with choroidal melanoma. Other studies have reached the opposite conclusion, where increased Beclin-1 expression in uveal melanoma has been associated with improved overall survival and a reduced risk of metastatic disease, highlighting the context-dependent role of autophagy in melanoma subtypes (Table 1) [105,107].

### 5.3. Molecular Biomarkers of Autophagy in Melanoma

Most autophagy-related regulatory proteins are not exclusively specific to the autophagic pathway. Therefore, reliable assessment of autophagic activity requires the analysis of multiple biomarkers evaluated in an appropriate temporal and biological context. Core autophagy-regulating proteins are frequently used as indicators of autophagic activity. For example, endogenous expression of the LC3B isoform has been associated with melanoma progression and unfavorable prognosis. Another potential biomarker in melanoma is Beclin-1. As is well known, studies have reported that Beclin-1 overexpression can lead to opposite effects; nevertheless, available data suggest a tendency toward reduced Beclin-1 expression in melanoma, with patients exhibiting low levels of this protein showing an increased likelihood of distant metastasis. In contrast, studies conducted in uveal melanoma have demonstrated that higher Beclin-1 immunohistochemical expression is associated with a lower risk of metastasis and prolonged recurrence-free survival. Conversely, reduced Beclin-1 protein expression in patients with uveal melanoma has been linked to an increased risk of metastatic disease (Table 1) [113,114,115].

Another marker used to evaluate autophagic activity is the level of p62 protein. Interestingly, increased p62 expression, rather than the expected reduction, may indicate autophagy inhibition due to the accumulation of protein aggregates containing p62. Notably, during the early stages of melanoma development, an increase in p62 expression has been observed, followed by its reduction in advanced metastatic tumors, which is consistent with autophagy reactivation and reflects its complex, context-dependent role in cancer progression. Moreover, tumors characterized by low p62 expression have been associated with an increased risk of metastasis (Table 1) [110,115,116].

Additionally, reduced ATG5 protein levels have been observed in early-stage melanoma, suggesting decreased autophagic activity during the initial stages of tumor development. Overall, melanoma initiation appears to be accompanied by autophagy suppression, which may allow tumor cells to evade endogenous tumor-suppressive mechanisms. However, as the disease progresses, autophagic activity increases in response to the metabolic requirements of the tumor, contributing to unfavorable clinical outcomes [115]. In early-stage melanoma, prognostic markers include the expression levels of p62 and an activating molecule in Beclin-1-regulated autophagy protein 1 (AMBRA1) [103,109].

It should be emphasized that the expression levels of autophagy-associated markers do not necessarily reflect the actual level of autophagic activity. For instance, alterations in LC3 protein levels may result either from impaired autophagosome degradation or from mechanisms independent of autophagy. Consequently, the quantification of LC3-II at a single time point is insufficient to accurately assess autophagic flux. Reliable evaluation of autophagic activity therefore requires dynamic approaches, including the use of lysosomal inhibitors to monitor LC3-II turnover, fluorescence microscopy-based assays, and transmission electron microscopy [115].

**Table 1:** The key autophagy-related molecules in melanoma.

Molecule	Function	Effect on Autophagy	Mechanism of Action	Melanoma Subtype or Type of Cells	Therapeutic Potential	References
ATG5	monoallelic deletion or promoter methylation of gene	inhibition	reduction in autophagosome membrane elongation	cutaneous melanoma	Poor patient survival, particularly in primary tumors	[97,103,104]
ATG5	downregulation of gene expression	inhibition	bypass oncogene-induced senescence	melanoma cells (in vitro)	Facilitating of malignant transformation	[103,104]
ATG5	downregulation of gene expression	inhibition	related to deficiency of NRF1	cutaneous melanoma	Facilitating of malignant transformation in early stages	[104]
ATG5	downregulation of gene expression	inhibition	related to deficiency of NRF1	BRAFV600E-driven melanoma	Limiting the initiation of malignant transformation	[97]
ATG7	deletion of gene	inhibition	related to deficiency of NRF1	BRAFV600E-driven melanoma	Suppressing of tumor initiation	[97]
ATG7	deficiency of protein	inhibition	the complex interplay between autophagy, oncogenic signaling pathways, and cellular stress responses	melanoma with BRAF V600E mutation and PTEN loss	Inhibiting the development of melanoma and its subsequent progression	[11,103,105,112]
ATG7	deficiency of protein	inhibition	the ATG7 deficiency is sensed by p53, which subsequently activates apoptotic pathways	oncogene-primed melanocytes	Acceleration of the tumor formation process	[111]
Beclin-1	downregulation of gene expression	inhibition	bypass oncogene-induced senescence	cutaneous melanoma	Facilitating malignant transformation in early stages	[104]
Beclin-1	protein activation	activation	phosphorylation of the EGFR	BRAF-mutant M14 and WM793 CM cell lines	Development and resistance to targeted therapy	[106]
Beclin-1	upregulation of gene expression	activation	hypoxia and acidic microenvironmental conditions	uveal melanoma	Early metastases and poor prognosis	[105,107]
Beclin-1	upregulation of gene expression	activation	hypoxia and acidic microenvironmental conditions	uveal melanoma	A lower risk of metastasis and a longer disease-free survival time	[105,107]
LC3	downregulation of gene expression	inhibition	regulation of MITF expression	cutaneous melanoma	Facilitating malignant transformation in early stages	[104]
LC3B	upregulation of gene expression	activation	regulation of MITF expression	heavily pigmented melanoma cells	Enhanced metastatic potential and poor clinical prognosis	[109]
p62	downregulation of gene expression	inhibition	-	cutaneous melanoma	Increased risk of metastasis	[110,115,116]

## 6. Therapeutic Perspectives

The autophagy process plays a significant role in the pathogenesis and development of melanoma. This process can both support and inhibit the development of melanoma. Therefore, appropriate modulation of autophagy may contribute to improving melanoma treatment. There is known research that suggests the potential use of autophagy modulators in the treatment of melanoma. In the case of C32 and A375 amelanotic melanoma cell lines, the addition of an autophagy inhibitor—chloroquine (CQ) or 3-methyladenine (3-MA)—to trametinib treatment resulted in increased antitumor activity compared to monotherapy with trametinib. The main effect was observed as an antiproliferative action, the p44/42 protein levels and other intracellular changes. Additionally, changes in the levels of the LC3A/B and LC3B indicated inhibition of the autophagy process (Table 2) [117].

GNAQ and GNA11 (GNAQ/11) are homologues genes encoding Gα subunits of heterotrimeric G proteins (crucial for G protein-coupled receptor signaling). Mutations in GNAQ/11 occur in a mutually exclusive manner and are present in more than 90% of uveal melanomas. Studies have shown that the combined inhibition of Gα and autophagy or lysosome function results in enhanced cell death. The analyses were conducted on GNAQ/11 melanoma mutant cells in vitro (OMM2.5, MP41, OMM1 and MEL270 cell lines) and in vivo in a mouse model of metastatic uveal melanoma. Furthermore, as the above study demonstrated that the combination of trametinib with chloroquine or hydroxychloroquine increased cytotoxicity, inhibited tumor growth and significantly prolonged survival in the test organisms (Table 2) [118].

Studies have been conducted to improve the efficacy of BRAF V600 inhibitor therapy. These were non-randomized Phase I/II clinical trials called BAMM (BRAF, Autophagy, and MEK Inhibition in Melanoma), which evaluated the use of dabrafenib (a BRAF inhibitor) and trametinib (a MEK inhibitor) in combination with hydroxychloroquine (HCQ). In one of the studies, this combination yielded an 85–88% response rate in stage IV melanoma with a BRAF mutation and was well tolerated [119], whereas in another study using the same combination, the treatment was also well tolerated but resulted in an increase in the RR rate and failed to meet the success criteria for one-year progression-free survival (PFS), which was 48.2% (Table 3) [120,121]. The following combinations have also been tested for melanoma: HCQ + CPI-613, HCQ + vemurafenib and HCQ + trametinib (metastatic NRAS melanoma) (Table 3) [122]. Studies conducted on a xenograft derived from a human with NRAS-mutated melanoma indicated that the combination of trametinib and chloroquine led to tumor regression, unlike when the drugs were used alone. Moreover, mice treated with combination therapy did not show any weight loss, but experienced side effects such as hair loss and a facial rash, which subsided after the chloroquine dose was reduced to 25 mg/kg—a dose that continued to demonstrate efficacy when combined with trametinib (Table 2) [123].

In melanoma, the most commonly used autophagy inhibitors, such as chloroquine and 3-methyladenine are not pharmacologically specific [105,124]. Chloroquine and its derivatives, well known as traditional antimalarial drugs, they are lysosomotropic agents with potential clinical application. These agents neutralize the lysosomal pH and inhibit the activity of lysosomal hydrolases. They can attenuate most, if not all, membrane trafficking routes that terminate or transit at lysosomes. As it might seem, the cytotoxic effect of CQ is not dependent on autophagy, but related to lysosomal membrane permeabilization, which can trigger autophagy-independent cell death. Moreover, an increase in pH within acidic compartments sensitizes tumors to treatments by increasing the intracellular retention of certain chemotherapeutic drugs [103,105,125]. 3-methyladenine is a Class III PI3K inhibitor, which inhibits autophagosome formation [103,105,126]. Interestingly, 3-MA has been shown to induce autophagy under nutrient-rich conditions while retaining its ability to inhibit starvation-induced autophagy. This dual effect is attributed to the distinct temporal effects of 3-MA on Class I and Class III phosphoinositide 3-kinases (PI3Ks). Specifically, 3-MA exerts sustained inhibition of Class I PI3K, whereas its inhibitory effect on Class III PI3K is transient [126]. Depending on the specific cellular context, this substance may have an additional effect on other PI3K activities. 3-MA may interact with other kinases (p38 MAPK or c-Jun kinase) and affect other cellular processes, such as glycogen metabolism, lysosomal acidification, endocytosis, the mitochondrial permeability transition, and also inhibit proteolysis even in ATG5-deficient cells [124,125,126]. This inhibitor has low potency, and it is typically used at very high concentrations (about 10 mM) to inhibit the autophagy process in vitro in cellular assays [124,126].

Chloroquine and hydroxychloroquine, as drugs with lysosomotropic potential, raise the intralysosomal pH, thereby affecting overall lysosomal function and impairing the autophagic degradation of proteins. These substances were used for malaria prophylaxis and treatment, rheumatoid arthritis, and human immunodeficiency virus. What is more, CQ and HCQ also exhibit pleiotropic activity and may have harmful properties. An example includes direct interaction with the TLR ligand, rather than an effect on lysosomal pH [127,128]. Furthermore, CQ and its derivatives can induce NF-κB, target telomeric G-quadruplex and palmitoyl-protein thioesterase 1, enhance antigen presentation by dendritic cells through endolysosomal deacidification and signaling activation and promote macrophage M2-to-M1 polarization. It is interesting to note that chloroquine can also directly induce apoptosis in a manner specific to melanoma—a proapoptotic process that is PUMA-dependent but not lysosomal protease-dependent. Therefore, it should be borne in mind that the effects caused by chloroquine and its derivatives may result not only from the inhibition of autophagy, but also from other mechanisms, such as broadly defined lysosomal dysfunction, membrane transport disorders, or modulation of the immune response [103]. These substances are generally well tolerated; however, the use of CQ and HCQ is associated with toxicity in the eye and the occurrence of cardiomyopathy. Toxicity in the eye is also related to cumulative dose, which can be problematic with long-term use. HCQ derivatives are likely safer than CQ and are designed to maintain their therapeutic effects without side effects [103,124,127].

Rapamycin is a well-known immunosuppressive drug that works by inhibiting mTOR. This substance also activates autophagy. In studies conducted on the B16-F10 (B16) mouse melanoma model, rapamycin was shown to have anticancer activity, and its use led to reduced survival and the induction of apoptosis in B16 cells both in vitro and in vivo (C57BL/6 mouse model) (Table 2) [129]. Other studies using a xenograft model of melanoma—generated by subcutaneous transplantation of human A375 melanoma cells into the backs of immunodeficient mice—showed that rapamycin treatment inhibited angiogenesis and lymphangiogenesis in melanoma. This effect may help control metastasis and, as a result, prolong patient survival (Table 2) [130].

Nanotechnology-based vaccines, when combined with autophagy activators, play a significant role in cancer immunotherapy by enhancing the effectiveness of dendritic cell (DC) activation in the body. Studies indicate that a vaccine in the form of the synthesized NP-B-OVA nanoparticle, which delivers the autophagy activator peptide Beclin-1 and the OVA antigenic peptide, synergistically improves antigen presentation efficiency and significantly enhances the effect of cytotoxic T-cell, (CTL)-mediated immunotherapy in melanoma by activating autophagy and delivering antigenic peptides to dendritic cells. The analyses were conducted in vivo using a mouse model with B16-F10-OVA melanoma cells (Table 2) [131,132].

Another example is bio-ATTEC nanoparticles, which induce autophagy-dependent cell death through mitochondrial degradation. Studies have shown that the use of these nanoparticles can significantly increase the sensitivity of metastatic melanoma to chemotherapeutic prodrugs in mice with B16–F10 melanoma (Table 2) [132,133].

Other studies revealed that melanoma cell lines transfected with EGFP-LC3 and then treated with CCI-779 (an analogue of rapamycin, temsirolimus) and hydroxychloroquine exhibited a greater cytotoxic effect than therapy alone. Additionally, research studies show that in vitro treatment of melanoma cells with temsirolimus and hydroxychloroquine synergistically increases cell death, this may lead to better therapeutic results. The combination of temsirolimus with HCQ results in metabolic stress in cancer cells (Table 2) [106,134,135].

Natural killer (NK) cells are immune system cells responsible for destroying cells such as cancer cells. Studies show that targeting BECN1 and other autophagy genes (SQTSM1/p62, ATG5) or using an autophagy inhibitor (e.g., chloroquine) decreased melanoma development due to the response of NK cells. The increased infiltration is caused by a mechanism involving the secretion of high levels of CCL5 (pro-inflammatory cytokine) [136,137].

**Table 2:** Preclinical studies on the modulation of autophagy in melanoma.

Drug	Target/Pathway	Combination Therapy	Experimental Evidence	Main Outcome	References
Trametinib	Inhibitor MEK	CQ or 3-MA	C32 and A375 amelanotic melanoma cells	Antiproliferative action (decrease in p44/42 protein levels)	[117]
Trametinib	Inhibitor MEK	CQ or HCQ	GNAQ/11 melanoma mutant cells in vitro (OMM2.5, MP41, OMM1 and MEL270 cell lines) and in vivo in a mouse model of metastatic uveal melanoma	Increase cytotoxicity, inhibited tumor growth and significantly prolonged survival	[118]
Trametinib	Inhibitor MEK	CQ	A xenograft derived from a human with NRAS-mutated melanoma	Tumor regression	[123]
CCI-779	Inhibitor mTOR	HCQ	Melanoma cell lines (C8161, A2058, UACC903 and SK-MEL-2) transfected with EGFP-LC3	Inhibition of melanoma growth and induction of cell death	[134]
Rapamycin	Inhibitor mTOR	-	B16 melanoma cells in vitro and in vivo in C57BL/6 mouse model	Reduced survival and induction of apoptosis	[129]
Rapamycin	Inhibitor mTOR	-	A xenograft model of melanoma (transplantation of human A375 melanoma cells into mice)	Inhibition of angiogenesis and lymphangiogenesis may help control metastasis and, as a result, prolong patient survival	[130]
Beclin-1	Autophagy activator peptide	Vaccine in the form of a synthetic NP-B-OVA nanoparticle that delivers the Beclin-1 peptide and the OVA antigenic peptide	A mouse model with B16-F10-OVA melanoma cells	Improving antigen presentation and significantly enhancing the effect of cytotoxic immunotherapy mediated by T cells	[131,132]
bio-ATTEC nanoparticles	Induction of autophagy-dependent cell death through mitochondrial degradation	-	Mice with B16–F10 melanoma	Increasing the sensitivity of metastatic melanoma to chemotherapeutic prodrugs	[132,133]

**Table 3:** Clinical studies on the modulation of autophagy in melanoma.

Drug	Target/Pathway	Combination Therapy	Clinical Evidence and Number of Participants	Main Outcome	References
Trametinib + dabrafenib	Inhibitor MEK + inhibitor BRAF	HCQ	Phase I/II clinical trials (NCT04527549) (*n* = 34)	The result did not meet the success criteria for the annual PFS ratio	[121]
Trametinib + dabrafenib	Inhibitor MEK + inhibitor BRAF	HCQ	Phase I/II clinical trials (NCT02257424) (*n* = 34)	This combination resulted in an 85–88% response rate in stage IV BRAF mutant melanoma and was tolerable	[119]
CPI-613 (devimistat)	Mitochondrial metabolism inhibitor	HCQ	Phase I/II clinical trials (NCT04593758)	Study results have not been submitted	[122]
Vemurafenib	Inhibitor BRAF	HCQ	Phase I clinical trials (NCT01897116)	Study results have not been submitted	[122]
Trametinib + dabrafenib	Inhibitor MEK + inhibitor BRAF	HCQ	Phase I/II clinical trials (NCT03754179)	Study results have not been submitted	[122]
Trametinib	Inhibitor MEK	HCQ	Phase Ib/II clinical trials (NCT03979651)	Study results have not been submitted	[122]

## 7. Conclusions

In summary, melanoma remains a highly aggressive malignancy and continues to represent a major public health challenge. Despite substantial advances in therapeutic strategies, including targeted therapies and immune checkpoint inhibitors, treatment resistance and suboptimal clinical responses remain significant obstacles to successful disease management. Increasing evidence indicates that autophagy plays a pivotal role in melanoma pathogenesis, although its function is highly context-dependent.

Autophagy exerts a dual role during melanoma development, acting as a tumor-suppressive mechanism during the early stages of tumorigenesis while promoting tumor survival, progression, and therapeutic resistance in advanced disease. In cutaneous melanoma, autophagy generally limits tumor initiation in stages I and II by maintaining cellular homeostasis and genomic integrity. In contrast, during advanced stages (III and IV), enhanced autophagic activity supports melanoma cell adaptation to metabolic stress, thereby facilitating tumor progression and resistance to anticancer therapies.

The role of autophagy in uveal melanoma appears to be similarly complex. Most studies associate increased autophagic activity with tumor progression, metastatic potential, and poor clinical outcomes. However, some reports suggest that reduced autophagic activity may also contribute to disease progression, highlighting the context-dependent nature of autophagy and the need for further investigation into its molecular regulation in this malignancy.

The impact of autophagy is further influenced by the genetic background of melanoma. For example, in BRAF-mutant melanoma, autophagy has been implicated in supporting tumor initiation and promoting resistance to targeted therapies. Conversely, in BRAF V600E melanomas with concurrent PTEN loss, impaired autophagy has been associated with accelerated tumor progression, emphasizing that the biological consequences of autophagy depend on the underlying molecular context.

Given its multifaceted role, modulation of autophagy has emerged as a promising therapeutic strategy for overcoming treatment resistance. Both pharmacological inhibition and induction of autophagy have demonstrated antitumor activity in preclinical models, suggesting that the therapeutic benefit of autophagy modulation may depend on the autophagic status and molecular characteristics of individual tumors. In particular, autophagy inhibitors, such as chloroquine and hydroxychloroquine, have shown encouraging results in combination with conventional and targeted therapies. Conversely, several autophagy-inducing agents have also been reported to suppress melanoma growth and promote cancer cell death under specific experimental conditions.

Collectively, the currently available evidence indicates that autophagy represents a promising, yet highly complex, therapeutic target in melanoma. A deeper understanding of the molecular mechanisms governing autophagy in distinct melanoma subtypes and genetic backgrounds will be essential for identifying patients who are most likely to benefit from autophagy-targeted interventions and for optimizing future therapeutic strategies.

## Data Availability

No new data were created or analyzed in this study. Data sharing is not applicable to this article.

## Abbreviations

The following abbreviations are used in this manuscript:

CMA	chaperone-mediated autophagy
ULK1	UNC-51 like kinase 1
ESCRT	endosomal sorting complex required for transport
ILV	intraluminal vesicle
SNARE	soluble N-ethylmaleimide-sensitive factor attachment protein receptors
BIF-1	Bax-interacting factor-1
RAS	rat sarcoma virus
AKT	protein kinase B
Bcl-2	B-cell lymphoma 2
UVRAG	ultraviolet irradiation resistance-associated gene
TLR	toll-like receptor
NF-κB	nuclear factor kappa-light-chain-enhancer of activated B cells
PTEN	phosphatase and tensin homolog
TSC1/2	tuberous sclerosis protein 1 and 2
DAPk	death-associated protein kinase
ROS	reactive oxygen species
NRF1	nuclear respiratory factor
RTK	receptor tyrosine kinase
EGFR	epidermal growth factor receptor
CREB	cAMP-responsive element binding protein
MITF	microphthalmia-associated transcription factor
mTOR	mammalian target of rapamycin complex
CAF	cancer-associated fibroblast
CTLA-4	cytotoxic T-lymphocyte antigen-4
PD-1	programmed cell death-1
NLR	NOD-like receptor family
TME	tumor microenvironment
